# Salivation Produced by Mixing Amalgams in the Hands

**Published:** 1874-04

**Authors:** 


*Salivation Produced by Mixing Amalgams in the Hands.*—An English dentist relates the case of his son who complained of tenderness in his gums, "increasing after an amalgam operation," together with the characteristic metallic taste in the mouth. The operator must be in the habit of using a large quantity of amalgam in his practice to produce such a result, or be almost as susceptible to the influence of mercury as the person in the case related by Dr. Watson in his Practice of Medicine, "that when his wife had rubbed a very small quantity of white precipitate ointment upon her neck, for some cutaneous affection, his gums were tender for three or four days after sleeping with her, and slight salivation took place. This did not happen once only, but three several times."

